# WSTF deficiency reprograms regulatory networks by linking locus–specific chromatin remodeling to altered isoform expression and misdirected signaling

**DOI:** 10.1093/nar/gkag602

**Published:** 2026-06-08

**Authors:** Shahin Behrouz Sharif, Jonathan H Dennis

**Affiliations:** Department of Biological Science, Florida State University, Tallahassee, FL 32306-4295, United States; Department of Biological Science, Florida State University, Tallahassee, FL 32306-4295, United States

## Abstract

Loss of the chromatin remodeler WSTF (BAZ1B), a gene deleted in Williams syndrome, causes reproducible, genome-scale reprogramming of chromatin states and transcript processing that links altered chromatin composition to misprocessed transcripts and aberrant signaling. Using engineered HCT116-WSTF^KO^ cells, we combine transcriptome profiling, microscopy, chromatin CUT&RUN, and histone post-translational modification (HPTM)-defined chromatin state modeling to show that WSTF localizes to promoters and gene bodies of actively transcribed loci together with ASH2L and CBP. Loss of WSTF causes depletion of ASH2L/CBP, selective loss of H3K4me2 and multiple acetylation marks, and gain of Polycomb components. This loss results in systematic conversion from a multi-mark active promoter/enhancer chromatin landscape to a hypoacetylated, H3K4me2-depleted, PRC-enriched landscape. These chromatin changes coincide with widespread isoform switching and splicing alterations in genes encoding chromatin regulators and signaling pathways. This WSTF deficiency produces Wnt/β-catenin hyperactivation. A locus-specific example at TCF7L2 demonstrates how gene body loss of active marks drives isoform switching that alters DNA binding domains and decouples stabilized nuclear β-catenin from canonical target engagement. Our results establish a mechanistic chain from WSTF-dependent chromatin architecture to co-transcriptional RNA processing integrity, signaling pathway fidelity, and developmental gene program regulation relevant to Williams syndrome.

## Graphical Abstract

**Graphical Abstract:**
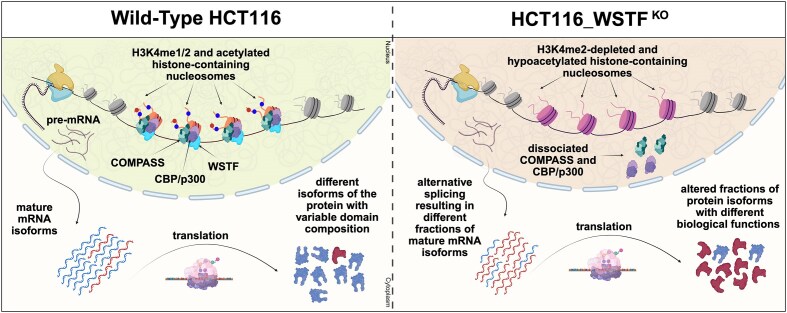

## Introduction

Williams Syndrome Transcription Factor (WSTF), also known as Bromodomain Adjacent to Zinc finger domain 1B (BAZ1B), is a multifunctional chromatin regulator with roles across the genome [1, 2]. WSTF lies within the recurrent microdeletion region at 7q11.23 that causes Williams–Beuren syndrome (WS), a contiguous–gene disorder that primarily affects cardiovascular and connective tissues and the central nervous system development [3, 4]. WSTF is critical for proper neural development: its knockdown in Xenopus laevis impairs neural crest migration, development, and survival, effects that are attributable to dysregulation of key developmental gene programs [5].

WSTF associates with the SNF2–related chromatin remodeling ATPase hSNF2H (SMARCA5) to form an ATP–dependent ISWI complex termed WICH (WSTF–associated ISWI CHromatin) that is involved in multitude of nuclear functions [6]. ISWI remodelers are involved in nucleosome assembly, spacing, and higher–order chromatin organization. The WICH complex is important for DNA replication, transcription, and repair; its depletion disrupts chromatin assembly immediately after DNA replication and produces smaller nuclei [7]. WSTF also participates in the larger B–WICH assembly, which is implicated in transcriptional regulation [8]. Loss of WSTF impairs transcription, likely through mis-regulated chromatin structure and defective recruitment of histone–modifying activities.

WS is primarily a developmental syndrome and the Wnt/β–catenin signaling pathway, a central developmental pathway that governs neural crest specification, migration, and cardiovascular morphogenesis processes, is disrupted in this syndrome. WSTF has been implicated as a regulator of canonical Wnt pathway fidelity as modulation of WSTF levels can alter Wnt pathway activity [9]. Wnt signaling integrates transcription factor recruitment through sequestered β–catenin in the nucleus to specific target genes. Perturbations in chromatin architecture caused by WSTF deficiency thus can alter biochemical pathway activation, for example, with increased nuclear β–catenin producing non–canonical outputs that may contribute to WS phenotypes.

Here we define WSTF’s contribution to the chromatin landscape and trace how its loss produces multilayered transcriptome rewiring. We show that WSTF stabilizes a multi–mark active chromatin signature whose collapse alters splicing pattern, isoform fraction, and transcription factor composition, mechanisms that can reprogram developmental and signaling pathways relevant to WS.

## Materials and methods

### Model cell generation

HCT116 cells (ATCC, CCL–247) were maintained in DMEM/F–12 (Sigma–Aldrich #D8900) supplemented with 10% FBS (Gibco A56707-01) and 1% Penicillin–Streptomycin in a 5% CO_2_ incubator at 37°C. CRISPR–Cas9 was used to delete the entire WSTF locus (∼82 kb) from both alleles at 7q11.23 (Supplementary Fig. S1). Two pairs of guide RNAs (gRNAs) were used to delete alleles sequentially. gRNAs were cloned into pX458 (Addgene #48138) and propagated in XL1–Blue competent cells (Agilent #200249). A pair of gRNAs targeting the upstream (5′) and downstream (3′) regions of the WSTF locus was transfected into proliferating HCT116 cells using Promega FuGENE^®^ HD (E2311). The resulting heterozygous WSTF knockout population was subjected to a second round of Cas9 deletion with a different gRNA pair using the same procedure. Single–cell colonies were FACS–sorted and screened by conventional PCR using primers that anneal outside the deletion region; amplification is possible only if the WSTF locus has been removed and the primer sites are brought into close proximity to yield an amplicon. Homozygous WSTF deletion was confirmed at the protein level by immunofluorescence and western blotting using anti–WSTF mouse monoclonal and rabbit polyclonal antibodies, respectively (Supplementary Table S1).

### Immunofluorescence staining

Approximately 1 × 10^5^ cells were seeded on poly–L–lysine–coated glass slides and incubated overnight. Fixation and permeabilization were performed in a single step with 0.1% Triton X–100 in 1× PBS containing 5% formaldehyde for 10 min at room temperature (RT). Slides were rinsed three times with PBS and blocked with 3.5% bovine serum albumin (BSA) in 1× PBS with 0.1% Tween–20 for 30 min at RT. Cells were incubated with primary antibody for 1 h at RT, washed three times with PBS, and incubated with the appropriate fluorescent secondary antibody for 30 min at RT. After a final PBS wash, nuclei were counterstained with DAPI and slides were imaged on an Olympus IX71 fluorescence confocal microscope.

### Expression and purification of pAG–MNase

The pAG–MNase expression plasmid (Addgene #123 461) was expressed in *Escherichia coli* JM101. Cultures were induced with 0.5 mM isopropyl β-D-1-thiogalactopyranoside (IPTG) and incubated at 16°C overnight. Cells were harvested and resuspended in extraction buffer, then sonicated for 15 cycles of 10 s on/10 s off at 80% power. Lysates were clarified and incubated with 5 ml nickel–NTA resin overnight. Resin was washed with extraction buffer and protein was eluted with elution buffer (0.1 M sodium phosphate, pH 8.0; 300 mM NaCl; 100 mM imidazole). Purified pAG–MNase was assayed for nuclease activity on chromatin prior to use in CUT&RUN.

### Chromatin mapping

Cleavage Under Targets and Release Using Nuclease (CUT&RUN) was performed essentially as described by Skene and Henikoff [10]. For each reaction, approximately 2.5 × 10^5^ intact cells were crosslinked with 0.1% formaldehyde for 2 min and quenched with 125 mM glycine (pH 2.0). Cells were immobilized on Concanavalin A–coated magnetic beads (BioMag^®^ Plus Concanavalin A, Polysciences Inc. #86057), washed in Wash Buffer containing 0.1% Triton X–100, and incubated with primary antibody or normal rabbit IgG overnight at 4°C. Excess antibody was removed by washing, and pAG–MNase was added to bind chromatin–associated antibody. MNase was then activated to cleave and release DNA fragments. Retrieved DNA fragments were prepared for sequencing using the NEBNext^®^ Ultra^™^ II DNA Library Prep Kit for Illumina^®^ (NEB #E7103) and sequenced (150 bp paired–end) on an Illumina NovaSeq X Plus to a depth of 10–15 million reads per library. All CUT&RUN experiments were performed on technical replicates of wild-type HCT116 cells and on two independent single cell colonies of HCT116-WSTF^KO^ biological replicates.

### Transcriptome analysis

Total nuclear RNA was isolated from biological and technical replicates using the NucleoSpin RNA Plus Mini kit with on–column DNase treatment (Macherey–Nagel, 740984). Polyadenylated RNA was enriched using the NEBNext^®^ Poly(A) mRNA Magnetic Isolation Module (NEB #E7490). Libraries were prepared with the NEBNext^®^ Ultra^™^ II Directional RNA Library Prep Kit for Illumina^®^ (NEB #E7765) and sequenced (150 bp paired–end) on an Illumina NovaSeq X Plus to a depth of ∼80 million reads per library.

### Western blotting

Approximately 5 × 10^6^ cells were washed in PBS containing Pierce^™^ Protease Inhibitor (ThermoFisher A32965) and lysed in 100 µl of 1× RIPA buffer (50 mM Tris–HCl pH 7.5, 150 mM NaCl, 1% NP–40, 0.5% sodium deoxycholate, 1 mM EDTA, and 0.1% SDS) supplemented with protease inhibitors. Lysis was performed on ice for 30–60 min. Lysates were clarified by centrifugation and protein concentration was measured by BCA assay (ThermoFisher PRO#23227). Approximately 30 µg protein per lane was resolved on 4%–20% Mini–PROTEAN^®^ TGX Stain–Free^™^ gels (Bio–Rad 4568094) and transferred to 0.22 µm Polyvinylidene Fluoride (PVDF) membranes. Membranes were incubated with primary antibodies overnight at 4°C, followed by HRP–conjugated goat anti–mouse or goat anti–rabbit secondary antibodies for 1 h at RT. Blots were developed using ECL and imaged on a Bio–Rad ChemiDoc system.

### Bioinformatic analysis

Raw FASTQ files were trimmed with Trimmomatic (https://github.com/usadellab/Trimmomatic) to remove adapters and low–quality bases. CUT&RUN and other DNA sequencing reads were aligned to the GENCODE GRCh38 reference genome using Bowtie2 (https://github.com/BenLangmead/bowtie2). Alignments were processed with SAMtools (https://github.com/samtools); reads with mapping quality < 10 and PCR duplicates were removed. Library complexity and coverage were assessed, and replicate concordance was evaluated by Spearman correlation and principal component analysis (PCA). Peaks were called with MACS3 (https://github.com/macs3-project/MACS) using broad peaks for histone post–translational modifications (HPTMs) and narrow peaks for chromatin remodelers and enzymes; a common IgG control was used for all samples and a *q*–value threshold of 0.01 was applied. BedGraph files were generated for genome browser visualization and heatmap production using deepTools (https://github.com/deeptools/deepTools).

For RNA–seq, reads were aligned with HISAT2 (https://github.com/DaehwanKimLab/hisat2) to GENCODE GRCh38. Low–quality reads (<10) and PCR duplicates were removed. Read summarization to genomic features was performed with featureCounts (https://github.com/gih0004/RNA_Seq_featurecounts) and differential expression analysis was conducted with DESeq2 (https://github.com/thelovelab/DESeq2). For splicing and isoform analyses, exon–exon junctions were detected using the subjunc function from the Subread package (https://github.com/ShiLab-Bioinformatics/subread). Transcript quantification was performed with Salmon (https://github.com/COMBINE-lab/salmon), differential exon usage was assessed with DEXSeq (https://github.com/dozmorovlab/DEXseq), and isoform switching was analyzed with IsoformSwitchAnalyzeR (https://github.com/kvittingseerup/IsoformSwitchAnalyzeR). All statistical analyses and figures were generated in R version 4.4.3.

## Results

### Loss of WSTF produces genome-wide transcriptional changes linked to Williams syndrome biology

To dissect the chromatin and transcriptome consequences of WSTF loss, we used HCT116-WSTF^KO^ cell lines. HCT116 cells were chosen because they are highly amenable to precise genome editing, have a complex transcriptome, and provide a well–characterized chromatin landscape for dissecting WSTF function.

**Figure 1.**
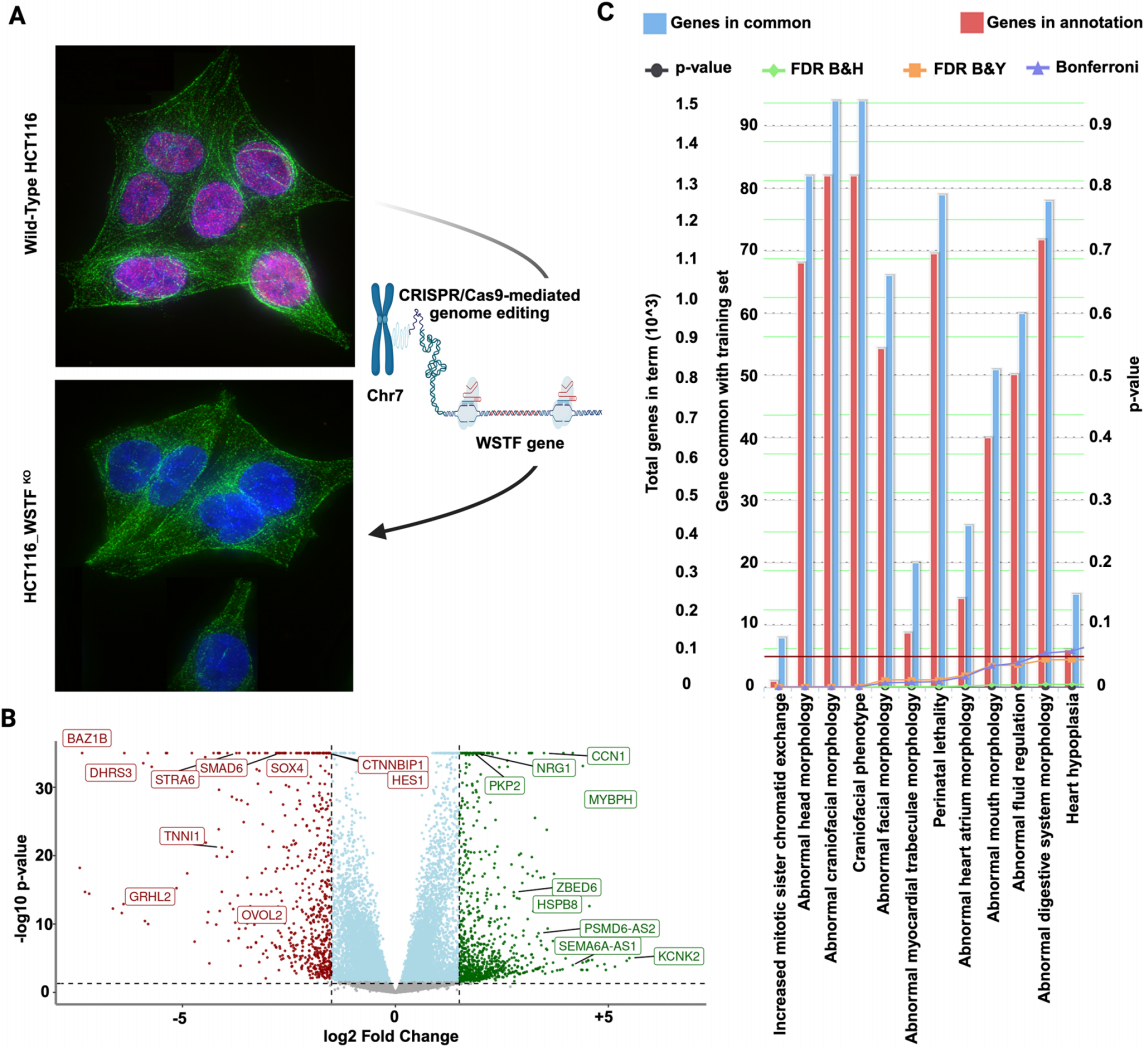
WSTF loss produces genome-wide transcriptional changes linked to Williams syndrome biology. (**A**) Immunofluorescence confirmation of WSTF knockout in HCT116 cells. Wild-type (WT) and HCT116-WSTF^KO^ cells were stained for WSTF (red), β-tubulin (green), and DAPI (blue). Complete loss of WSTF nuclear signal confirms homozygous deletion. (**B**) Volcano plot of differential gene expression in HCT116-WSTF^KO^ versus WT cells. Each point represents one gene. Horizontal dashed line: adjusted *P*-value = 0.05; vertical dashed lines: |log2FC| = 1. Genes exceeding both thresholds are highlighted. Approximately 1500 genes are differentially expressed, enriched for transcription factors, signaling components, and regulators of neural crest and cardiovascular development. (**C**) Mouse-phenotype terms enriched among DEGs in HCT116-WSTF^KO^ cells, identified using the ToppGene suite (https://github.com/ImmuSystems-Lab/toppgene). Each term is shown as a paired bar chart: blue bars indicate the number of DEGs overlapping the annotation term (left axis); red bars indicate the total number of genes in the annotation term (left axis). Overlaid lines show the *P*-value under four multiple-testing corrections (raw *P*-value, FDR Benjamini–Hochberg, FDR Benjamini–Yekutieli, and Bonferroni; right axis). Terms are ordered by enrichment significance. Enrichment for abnormal craniofacial morphology, abnormal head morphology, craniofacial phenotype, abnormal cardiac trabeculae morphology, and heart hypoplasia connects the HCT116-WSTF^KO^ transcriptional program to the principal clinical features of Williams syndrome through pathway-level evidence.

We profiled and compared the transcriptome of HCT116-WSTF^KO^ to its parental cells to define the scope of gene expression changes following WSTF loss. Differential expression analysis identified ∼1500 genes with changes greater than ∼2.8–fold, indicating broad regulatory consequences of WSTF depletion (Fig. 1B and Supplementary Table S2). The affected genes are enriched for transcription factors and signaling components and map to pathways controlling cell proliferation, migration, development, and signaling cascades such as canonical Wnt/β–catenin, suggesting that primary chromatin defects propagate into major regulatory networks.

Among the mis–expressed genes we observed an enrichment for regulators of neural crest development (e.g. FGF19, NRG1, SEMA6A/B, ISL1, NRP1/2, HES1, and OVOL1/2), consistent with WSTF’s established role in neural crest biology. Genes involved in heart and cardiovascular development were also dysregulated despite the epithelial origin of HCT116 cells. Specifically, we detected 34 differentially expressed genes (DEGs) linked to cardiac chamber, septum, and ventricular development: SMAD3, CNN1, HEG1, PKP2, KCNK2, and NOTCH2 were upregulated, whereas SMAD6, BMP5, TNNI1, TNNI3, and MESP1 were downregulated. Mouse–phenotype enrichment analysis further associated the transcriptional signature with abnormal craniofacial and cardiac chamber morphology (Fig. 1C), reinforcing the developmental relevance of WSTF–dependent chromatin regulation.

These findings establish that WSTF loss drives a broad transcriptional program linked to the principal clinical features of Williams syndrome. The enrichment for neural crest, cardiac, and Wnt pathway genes among the DEGs in HCT116-WSTF^KO^ cells, together with the mouse-phenotype enrichment for craniofacial and cardiac morphology defects (Fig. 1C), connects the chromatin regulatory consequences of WSTF loss to the developmental pathways disrupted in WS patients. We use HCT116-WSTF^KO^ cells throughout this study as a tractable model for dissecting the chromatin mechanisms through which WSTF regulates transcription. We next characterized the cellular phenotypes arising from WSTF loss to directly link these transcriptional alterations to specific cellular outputs.

### WSTF depletion promotes hyperactivation of canonical Wnt signaling

As prior work showed that reintroduction of WSTF into WS–derived induced Pluripotent Stem Cells (iPSCs) can reduce Wnt hyperactivation [9], we asked whether loss of WSTF is sufficient to alter Wnt pathway activity in our HCT116 model. To test this, we monitored the nuclear pool of non–phospho–Ser45 (active) β–catenin, a widely used readout of stabilized, transcriptionally competent β–catenin [11]. HCT116 cells are well suited for this assay as they have and active and measurable signaling cascade and the nuclear accumulation of active β–catenin, which reflects the levels of pathway hyperactivation, can be quantified by immunofluorescence staining.

**Figure 2.**
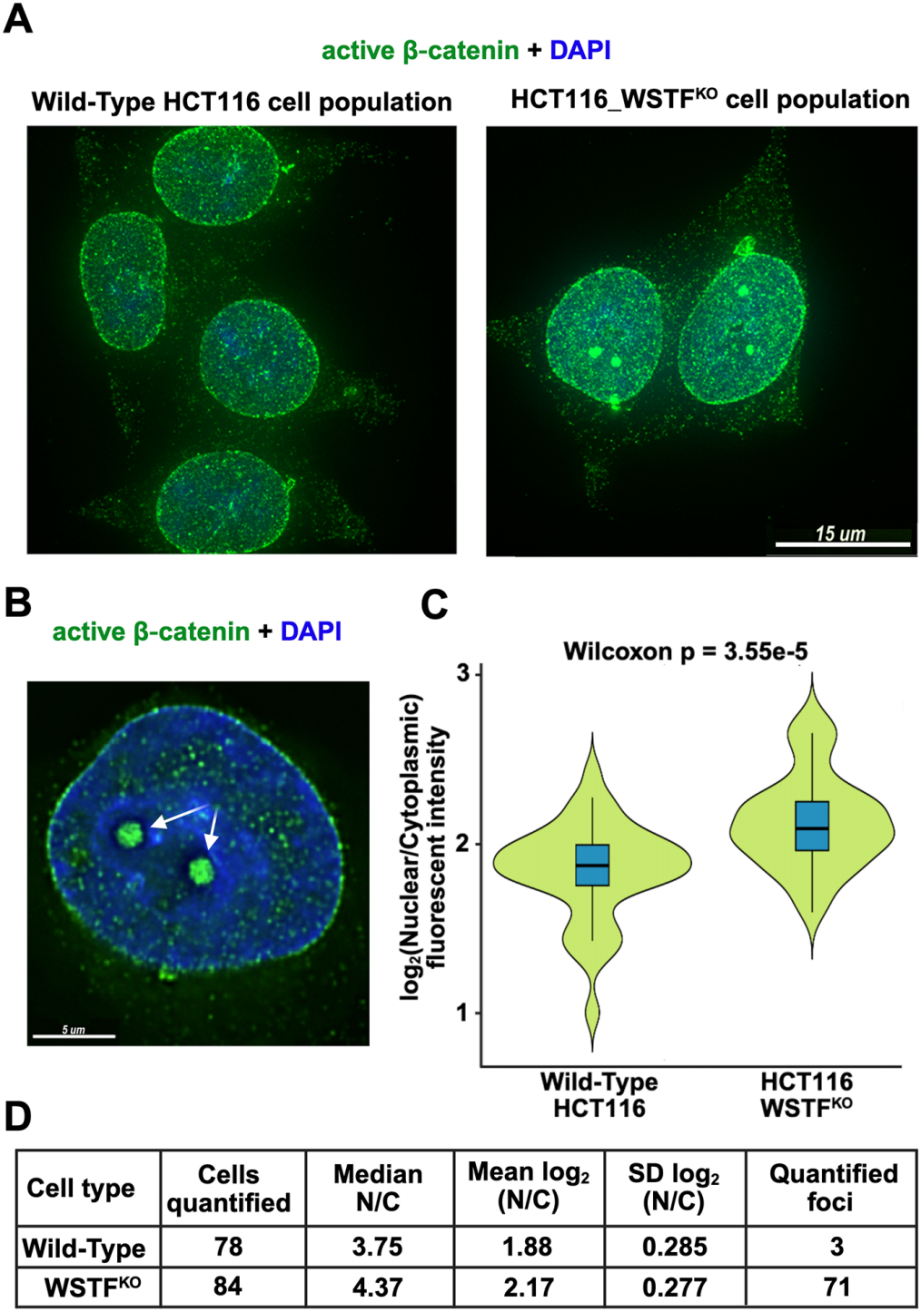
Hyperactivation of Wnt/β-catenin signaling in HCT116-WSTF^KO^ cells. (**A**) Immunofluorescence of interphase WT and HCT116-WSTF^KO^ cells stained for non-phosphorylated (active, non-phospho-Ser45) β-catenin under standard growth conditions without exogenous Wnt stimulation. Nuclei are counterstained with DAPI. Increased nuclear localization of active β-catenin is evident across the WSTF^KO^ population. (**B**) Representative single-cell image of a HCT116-WSTF^KO^ nucleus showing discrete nuclear foci. Foci were defined as discrete, DAPI-independent globular intensity maxima of the active β-catenin signal within the DAPI-masked nuclear region, identified with a fixed intensity applied uniformly across WT and knockout images. See supplementary Movie S1 for better visualization of a representative β-catenin aggregation. (**C**) Distribution of per-cell log2(*N/C*) ratios of active β-catenin immunofluorescence signal in WT (*n* = 78 cells) and WSTFKO (*n* = 84 cells) HCT116 cells, shown as a combined violin/box/jitter plot. *N/C* ratio was computed from background-corrected integrated density measurements; nuclear regions were defined by DAPI masks and cytoplasmic signal derived from whole-cell minus nuclear integrated density (see Supplementary Fig. S2). Violin shape shows the full distribution, and box shows median and interquartile range. WSTF^KO^ cells show a significant increase in nuclear enrichment (Wilcoxon rank-sum *P* = 3.55 × 10^−5^). (**D**) Summary statistics table for the quantitative immunofluorescence analysis shown in panel (C). For each cell type, the table reports the number of cells quantified (*n*), median *N/C* ratio, mean log2(*N/C*), and standard deviation of log2(*N/C*). WT: *n* = 78, median *N/C* = 3.75, mean log2(*N/C*) = 1.88, SD = 0.285. WSTFKO: *n* = 84, median *N/C* = 4.37, mean log2(*N/C*) = 2.17, SD = 0.277. The increase in mean log2(*N/C*) is significant by Wilcoxon rank-sum test (*P* = 3.55 × 10^−5^) and Welch *t*-test [*P* = 1.77 × 10^−5^; 95% CI for mean difference = 0.162–0.411 in log2(*N/C*)].

Using these readouts, we found that WSTF knockout drives constitutive nuclear accumulation of active β–catenin under standard growth conditions, in the absence of exogenous Wnt ligand (Fig. 2A). In HCT116-WSTF^KO^ cells, the nuclear signal is not only increased but often organized into multiple large nuclear foci, a consistent pattern of β–catenin aggregation, which is not associated with DNA and suggests abnormal nuclear sequestration of the pathway effector (Fig. 2B and Supplementary Movie S1). These results indicate that WSTF loss is sufficient to dysregulate Wnt signaling at the level of β–catenin stabilization and nuclear localization.

We quantified nuclear enrichment of active β-catenin on a per-cell basis by computing a background-corrected nuclear-to-cytoplasmic (*N/C*) intensity ratio from immunofluorescence images (Fig. 2C and D, and Supplementary Fig. S2). WSTF^KO^ cells show a significant increase in nuclear enrichment relative to wild-type cells (median *N/C* ratio: 3.75 [WT] versus 4.37 [WSTF^KO^]; mean log_2_(*N/C*): 1.88 ± 0.285 [WT, *n* = 78] versus 2.17 ± 0.277 [WSTF^KO^, *n* = 84]; Wilcoxon *P* = 3.5 × 10⁻⁵). As independent functional evidence that the elevated nuclear β-catenin pool is transcriptionally active, canonical Wnt target genes are significantly upregulated in WSTF^KO^ cells: CCND1 (Cyclin D1; log₂FC = +1.42, padj = 1.27 × 10⁻⁵³) and AXIN2, a negative-feedback target that serves as the most reliable transcriptional readout of active β-catenin/TCF signaling (log₂FC = +1.54, padj = 1.86 × 10⁻²^7^). TCF7L2 itself is also modestly but significantly upregulated (log₂FC = +0.38, padj = 1.05 × 10⁻⁶). Together, the quantitative imaging and transcriptional evidence confirm constitutive, transcriptionally competent Wnt pathway activation upon WSTF loss.

These observations suggest a direct link between WSTF–dependent chromatin regulation and control of Wnt pathway output. Loss of WSTF correlates with increased pools of active β–catenin in the nucleus, which can plausibly drive the transcriptional changes we observed genome–wide. Given the robustness of this phenotype in our model, we next sought to define the underlying chromatin structural defects that could causally connect WSTF loss to altered β–catenin dynamics and downstream gene mis–expression.

### WSTF localizes to promoters and gene bodies and promotes active chromatin states

**Figure 3.**
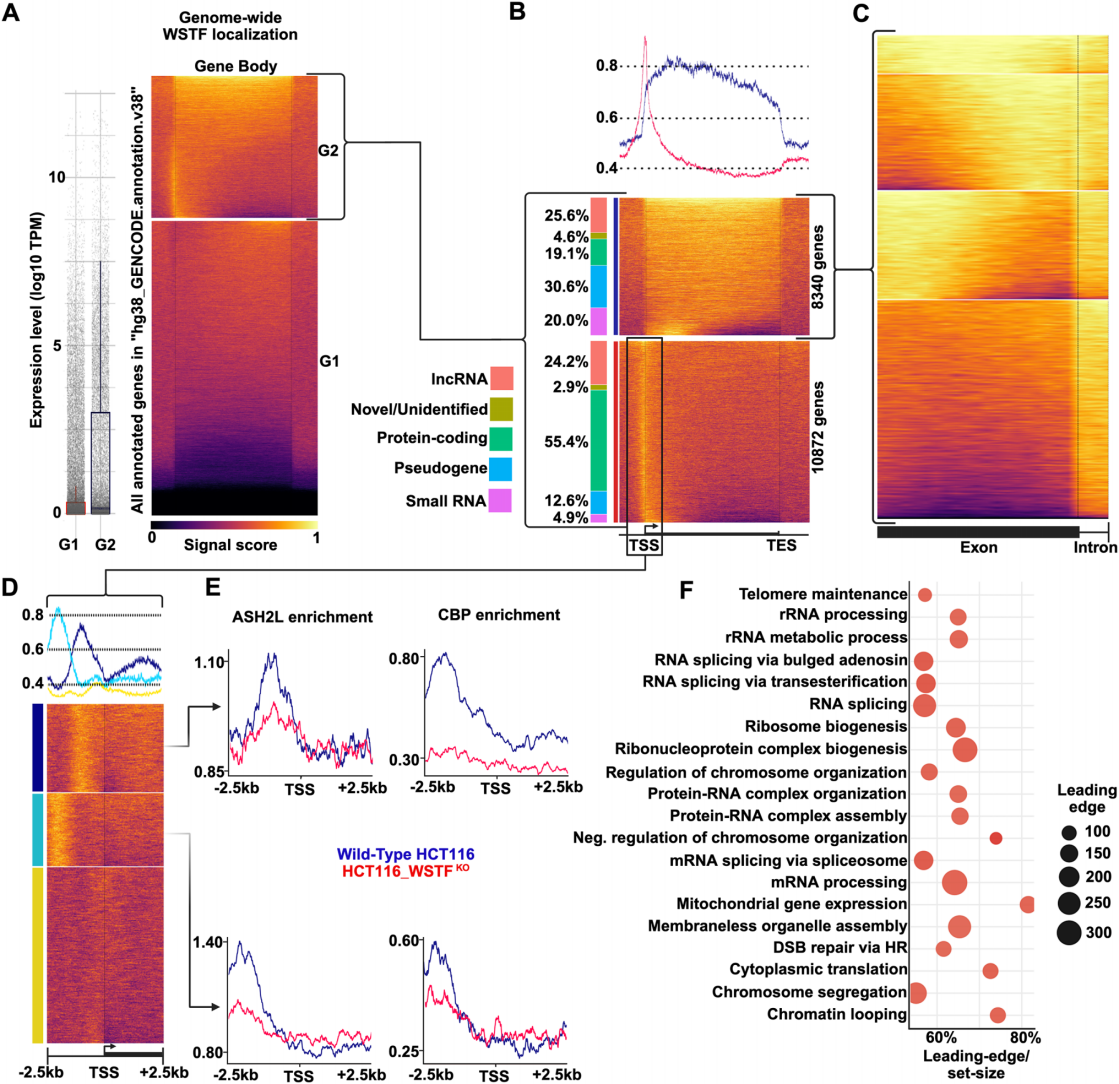
WSTF localization in HCT116 chromatin and gene ontology enrichment of highly WSTF-occupied genes. (**A**) Genome-wide WSTF binding profile in HCT116 cells determined by CUT&RUN. Signal is shown as normalized read depth across the genome, illustrating preferential enrichment at a highly active subset of loci. (**B**) Meta-gene profiles of WSTF CUT&RUN signal across open reading frames, scaled from transcription start site (TSS) to transcription end site (TES). Two dominant binding patterns are evident: enrichment concentrated at the promoter or distributed broadly across the gene body, defining the Promoter-enriched and Body-enriched occupancy classes used in panel (F). (**C**) WSTF distribution at exon–intron boundaries. Aggregate profiles are centered on splice donor and acceptor coordinates. Enrichment on exons flanking splice sites indicates preferential WSTF association with actively spliced chromatin regions. (**D**) WSTF localization at promoter regions. Profiles are centered on the TSS (±2 kb). Two dominant upstream binding patterns are observed: one peak close to the TSS and a second at a greater upstream distance, indicating positionally distinct modes of WSTF promoter association. (**E**) Co-localization of the two promoter-proximal WSTF patterns with ASH2L and CBP. Heatmaps show concordant enrichment of all three factors at promoters of actively transcribed genes, with WSTF occupancy flanked by ASH2L and CBP signal consistent with co-regulated chromatin states. (**F**) Pre-ranked gene set enrichment analysis for biological pathways (GSEA; GO:BP) based on promoter-proximal WSTF occupancy (±2.5 kb around TSS on strand-aware promoter windows) reveals significant enrichment (FDR < 0.05) for processes related to chromosome organization, rRNA processing as well as mRNA splicing and processing. Leading edge is the subset of genes in a gene set that are most highly correlated with a phenotype and contribute most to the enrichment signal.​ Bubble/dot plot shows the top enriched biological process terms. The *x*-axis shows the leading-edge fraction (leading-edge gene count divided by gene set size, expressed as a percentage); dot size encodes the absolute leading-edge gene count (scale: 100–300 genes); colour is uniform (coral). Terms are dominated by RNA processing and splicing functions: RNA splicing, mRNA splicing via spliceosome, mRNA processing, rRNA processing, rRNA metabolic process, ribosome biogenesis, ribonucleoprotein complex biogenesis, protein–RNA complex organization and assembly, and membraneless organelle assembly. Additional enriched terms include chromosome segregation, regulation of chromosome organization, DSB repair via homologous recombination, chromatin looping, and mitochondrial gene expression.

To define how WSTF contributes to chromatin architecture, we mapped its genome–wide distribution. Although WSTF has been implicated in multiple remodeling activities, the precise genomic contexts in which it acts remain unclear. Our CUT&RUN profiles show that WSTF occupies a distinct, highly active subset of genes (Fig. 3A) and displays two dominant enrichment patterns: it is either confined to promoter regions around the TSS or broadly distributed across gene bodies (Fig. 3B). Within gene bodies, the WSTF signal is biased toward a dynamic pattern on exonic regions relative to introns, suggesting a role in transcriptional events and co–transcriptional RNA maturation rather than simple promoter bookmarking (Fig. 3C). At promoters, WSTF localizes upstream of the TSS at two prominent positions (Fig. 3D). These distribution patterns led us to investigate how WSTF may accompany other chromatin–associated factors at the same loci.

We compared WSTF localization with key histone–modifying complexes. WSTF colocalizes with ASH2L and CBP at promoters (Fig. 3E). ASH2L is a core scaffold of mammalian COMPASS complexes that mediate H3K4 methylation [12], and CBP is a histone acetyltransferase that acetylates multiple residues on H3 and H4 [13]. This colocalization supports the involvement of WSTF in a coordinated program to establish transcriptionally permissive marks. Conversely, WSTF removal causes a pronounced loss of ASH2L and CBP occupancy at these loci, indicating that WSTF is required, directly or indirectly, for stable recruitment or retention of H3K4 methylation and histone acetylation activities. A pre-ranked gene set enrichment analysis (GSEA) of all those gene promoter regions (TSS ± 2.5 kb) that are targeted by WSTF reveals significant biological pathways enriched for processes related to chromosome organization, rRNA processing and mRNA splicing (Fig. 3F).

**Figure 4.**
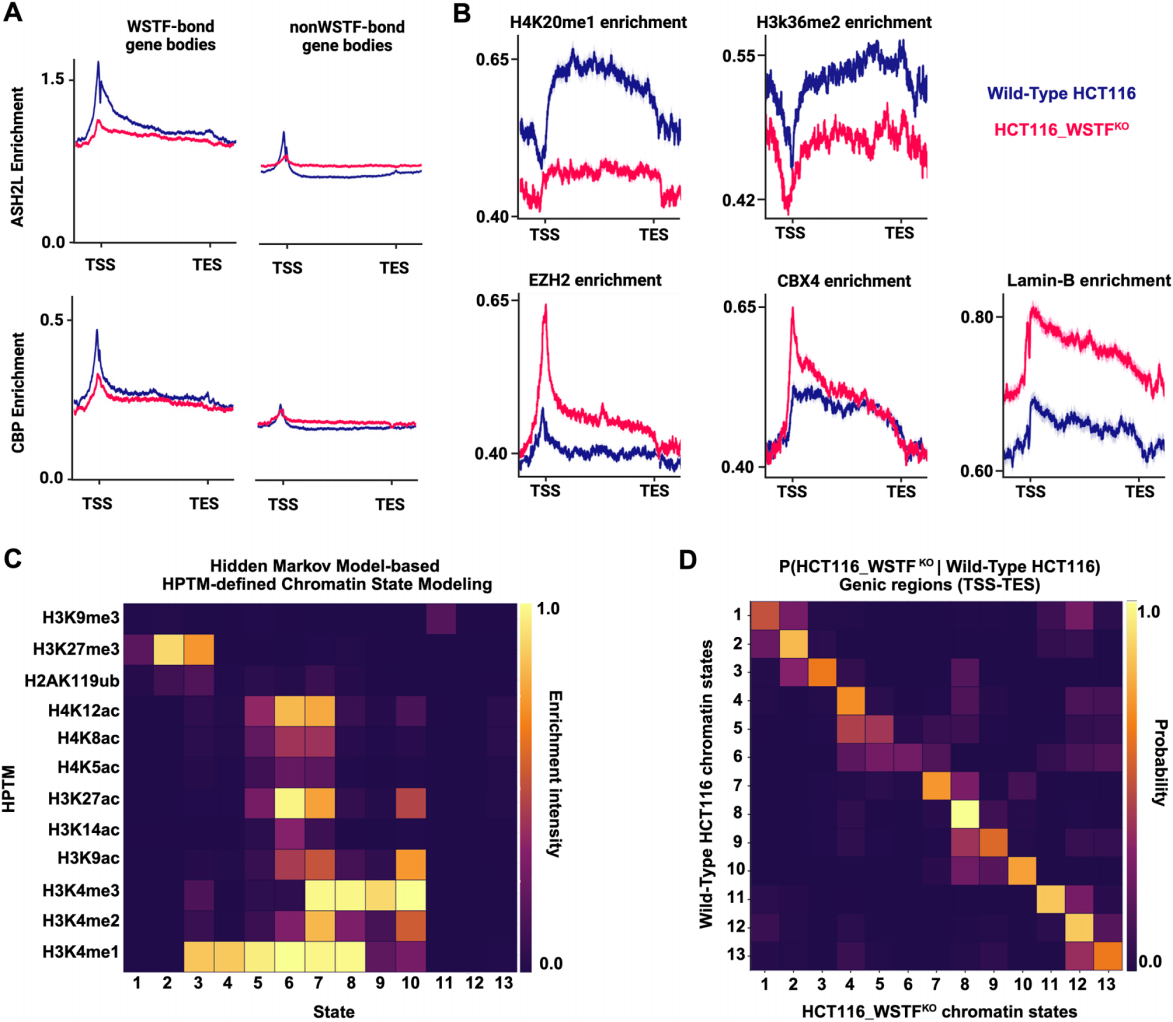
Genome-wide chromatin defects at WSTF-occupied genes following WSTF loss. (**A**) TSS-to-TES enrichment profiles for ASH2L and CBP illustrated for WSTF-bound gene bodies and non-WSTF gene bodies. The opposite effect of ASH2L and CBP enrichment alterations are shown for these group of genes. (**B**) Profiles of H4K20me1, H3K36me2, EZH2, CBX4, and Lamin-B in WT and WSTF^KO^ HCT116 cells, averaged over genes that WSTF associates with their body (defined in Fig. 3B). WT cells (blue lines) show prominent TSS-proximal peaks that extend into gene bodies at Body-enriched loci, along with broad enrichment of active elongation marks (H4K20me1, H3K36me2) across gene bodies. In WSTF^KO^ cells (red lines), Polycomb components EZH2 and CBX4 together with Lamin-B are gained across formerly active loci. These profiles provide direct evidence that WSTF loss collapses the multi-mark active chromatin state at its target genes. (**C**) ChromHMM 13-state chromatin model defined by combinatorial enrichment patterns of HPTMs in WT HCT116 cells. States are annotated by mark composition into functional categories including active promoter/enhancer, transcribed gene body, bivalent, Polycomb-repressed, heterochromatic, and quiescent states. (**D**) Probability heatmap comparing chromatin state assignments in WT versus WSTF^KO^ cells for each of the 13 ChromHMM states. Each cell shows the probability that a WT-annotated state transitions to each state in WSTF^KO^ cells. The most active WT states (active promoter, strong enhancer, transcribed gene body) show the greatest redistribution toward Polycomb-repressed and hypoacetylated states in WSTF^KO^ cells, formalizing the systematic conversion of active chromatin architecture illustrated in panel (A).

WSTF–bound gene bodies are associated with euchromatic marks and WSTF loss permits accumulation of repressive features. In addition to pronounced down-enrichment of ASH2L and CBP around TSS in WSTF depleted chromatin, gene bodies normally bound by WSTF show marked reductions in H4K20me1 and H3K36me2, marks associated with transcriptional activity and open chromatin. At the same time, Polycomb components (CBX4 of PRC1 and EZH2 of PRC2) display enhanced, coordinated localization across these gene bodies and promoters (Fig. 4A and B); a redistribution consistent with the antagonistic relationship between H3K36me2 and PRC2 activity. We also detected increased Lamin–B association at affected regions, suggesting altered nuclear topology that may reinforce a repressive environment.

To quantify how WSTF loss may alter the chromatin landscape genome–wide, we profiled twelve major HPTMs covering active marks (H3K4 methylation), Polycomb/heterochromatin marks (H3K27/K9 methylation and H2AK119ub), and multiple acetylation marks (H3K9/K14/K27 and H4K5/K8/K12). We integrated signal intensities in 200–bp bins using a multivariate Hidden Markov Model [14], which converged on 13 recurrent chromatin states ranging from promoter–like (states 3, 4, and 7–10) and enhancer–like (states 5 and 6) states to heterochromatin (states 1, 2, and 11) and quiescent (states 12, and 13) states (Fig. 4C). This state model provides a framework to comparef chromatin configuration at each locus between wild–type and WSTF^KO^ genomes.

Comparing state assignments between wild–type and WSTF^KO^ genotypes revealed a consistent theme, loss of WSTF produces a coordinated collapse of the active chromatin signature. Specifically, we observe concurrent loss of multiple histone acetylation marks together with selective down–enrichment of H3K4me2. For example, loci classified as wild–type state 10 are more likely to be re–established as states 8 or 9 in WSTF^KO^, which retain similar mark patterns but at reduced intensity and selective loss of H3K4me2 (Fig. 4D). These shifts indicate that impaired recruitment of ASH2L–dependent H3K4 methyltransferase complexes and CBP/p300 acetyltransferase leads to alternative, less active chromatin states characterized by a constellation of inter-related changes rather than isolated loss of a single mark.

These data suggest that WSTF stabilizes an active chromatin configuration by promoting recruitment of ASH2L– and CBP–dependent activities and by preventing inappropriate Polycomb engagement. Loss of WSTF therefore converts loci from an acetylated, H3K4–methylated state toward a more repressive, PRC–enriched configuration, providing a mechanistic link between WSTF occupancy and the transcriptional dysregulation observed in WSTF^KO^ cells.

### WSTF depletion drives widespread isoform switching

**Figure 5.**
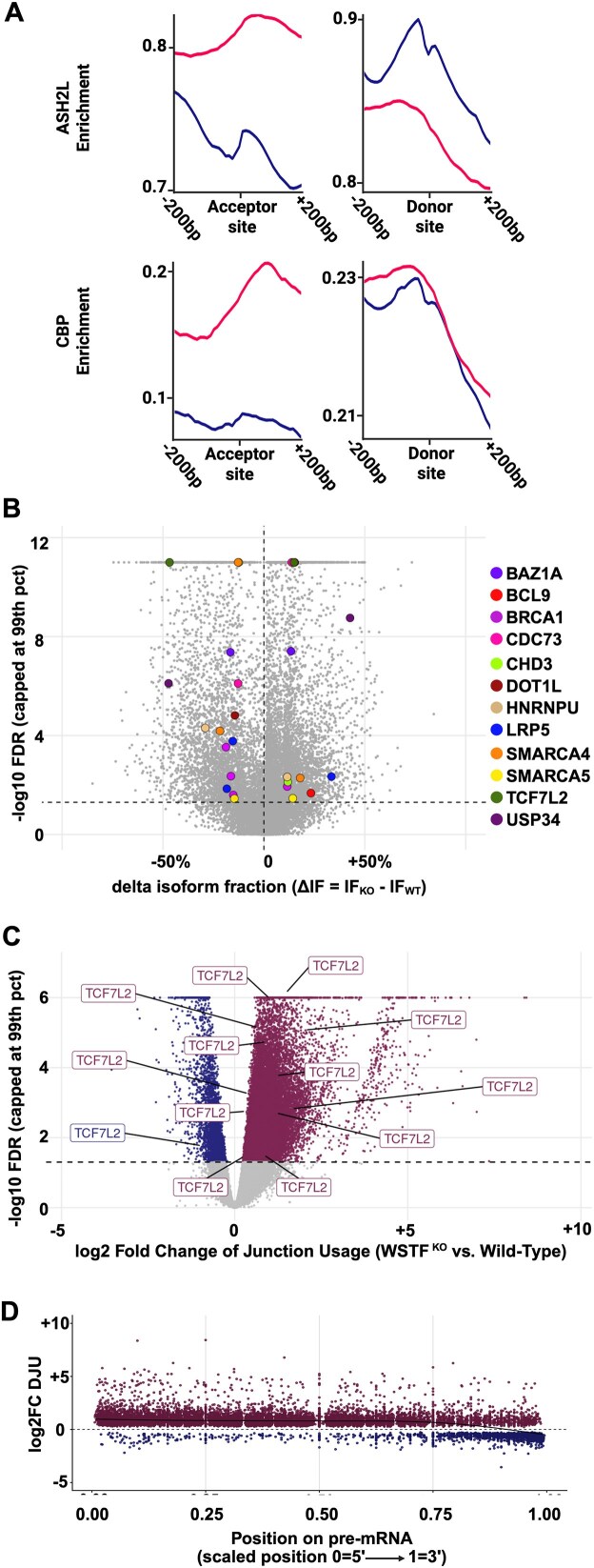
ASH2L and CBP redistribution at splice sites and isoform switching and splicing defects in the absence of WSTF. (**A**) Splice-site-anchored aggregate enrichment profiles for ASH2L (top row) and CBP (bottom row) in WT (blue) and WSTFKO (red) HCT116 cells. Profiles are generated from the same matrices used for the statistical analyses. Quantitative analysis of ASH2L and CBP enrichments across splice sites and their differences in WT and KO cells are presented in Supplementary Tables 5 and 6. (**B**) Differential isoform expression (isoform switching) in HCT116-WSTF^KO^ cells. The volcano plot shows isoform fraction change (ΔIF = IF^KO^ − IF^WT^) versus significance (−log10 FDR) for all tested transcripts. Among 16 322 tested transcripts, 5848 isoform switches affecting 2659 genes are detected at >10% ΔIF. Representative chromatin remodelers and canonical Wnt pathway genes are highlighted. (**C**) Differential splice-junction usage (DJU) in HCT116-WSTF^KO^ cells. Each point represents one splice junction; color indicates direction of change. The 13 TCF7L2 junctions are highlighted, illustrating that individual genes can contribute multiple significantly altered junctions with varied directionality. (**D**) Relative transcript-position distribution of differentially used splice junctions. Scaled position is defined as 0 (5′ end) to 1 (3′ end). Junctions with increased usage in WSTF^KO^ cells are enriched near the 5′ end of transcripts; junctions with decreased usage concentrate toward the 3′ end. This 5′-biased increase and 3′-biased decrease is a reproducible positional pattern that is not explained by local WSTF occupancy at splice sites.

Because chromatin alterations caused by WSTF loss extended beyond promoters and were enriched at exons, we asked whether these local changes influence co–transcriptional mRNA maturation. Such exon–localized chromatin changes could drive alternative isoform production, affecting protein function and RNA stability without altering gene–level expression. Given that WSTF normally associates with ASH2L and CBP, its loss is expected to lead into an unbalanced distribution of these factors, which are may regulate Pol-II behavior during transcription through depositing histone modifications [15]. Accordingly, we analyzed ASH2L and CBP using strand-aware matrices centered directly on donor (5′ splice site; exon end) and acceptor (3′ splice site; exon start) coordinates (±200 bp; 10-bp bins). Both ASH2L and CBP display specific pattern centered on the splice coordinates in WT genome, which are defective in WSTF^KO^ chromatin (Fig. 5A). All four panels show a peak centered on the splice coordinate in WT cells, confirming that ASH2L and CBP enrichment is genuinely junction-centered rather than restricted to exon ends. In WSTF^KO^ cells, both factors show reduced signal at first-exon donor sites (consistent with loss of promoter-proximal enrichment documented in Fig. 3E) and increased signal at internal donor and acceptor sites, indicating a redistribution of ASH2L and CBP away from gene 5′ ends toward internal splice-site neighborhoods upon WSTF loss. To quantify local enrichment, we computed a local boundary metric (peak_local) as the mean signal within ±20 bp of the splice coordinate, and quantified exon-side versus intron-side asymmetry using edge_delta = exon_edge - intron_edge (Supplementary Table S5). In WSTF^KO^ cells, both factors show reduced signal at first-exon donor sites (ASH2L: median delta(KO-WT) = −0.575, *P* < 1 × 10^−300^; CBP: median delta(KO-WT) = −0.025, *P* = 5.68 × 10^−24^), consistent with the loss of promoter-proximal enrichment documented in Fig. 3E, while simultaneously showing increased signal at internal donor and acceptor sites (ASH2L: median delta(KO-WT) = +0.15 at both internal donors and acceptors, *P* < 1 × 10^−300^; CBP: median delta(KO-WT) = +0.125 at internal donors, +0.10 at internal acceptors; Supplementary Table S6). This redistribution pattern, away from promoter-proximal first-exon sites and toward internal splice-site neighborhoods, suggests that WSTF loss does not simply lead in to reduced ASH2L and CBP occupancy uniformly but rather disrupts their normal spatial organization along the gene, which may contribute to the co-transcriptional splicing misregulation we observe genome-wide.

We interpret this as a consequence of altered nuclear protein homeostasis not as a direct mechanism via WSTF. When WSTF is lost, ASH2L and CBP that normally occupy WSTF-dependent sites are displaced. Rather than partitioning into background, the free nuclear pool of these factors redistributes, and a portion of the pool accumulates at loci where these factors would not normally be engaged, including internal splice sites. This redistribution is stochastic at the single-locus level but is reproducible in aggregate, and it is the expected outcome of a network-level perturbation of the kind WSTF loss represents.

To test the transcriptome fidelity, we quantified isoform fraction (IF), the proportion of a gene’s total expression contributed by each transcript and calculated the change in IF between genotypes (ΔIF). This metric reveals redistribution among alternative transcripts independent of net gene output. In HCT116-WSTF^KO^ cells, among 16 322 tested transcripts we detected 5848 isoform switches affecting 2659 genes (>10% ΔIF) (Fig. 5B and Supplementary Table S3). These results show that WSTF loss broadly redistributes transcript isoform usage, an observation we extend to a disease-relevant context in a dedicated section below.

Many isoform switches have functional consequences for RNA fate and coding potential. In HCT116-WSTF^KO^ cells, 586 switches created or removed a predicted termination codon (PTC), thereby altering transcripts’ susceptibility to nonsense–mediated decay (NMD). An additional 551 switches involved intron retention events that change the translated sequence of mature messenger RNAs (mRNAs). Gene ontology analysis of affected genes highlighted pathways for chromosome segregation and organization, RNA biogenesis (including splicing and localization), and heterochromatin formation. Notably, chromatin regulators such as BAZ1A, BRCA1, CENPV, EHMT2, EZH1, EZH2, HNRNPU, and SMARCA4 are among the genes with altered isoform architectures, suggesting a feed–forward vulnerability in which chromatin disruption induces mis-splicing of chromatin regulators and amplifies epigenomic instability.

To directly measure splicing changes, we quantified splice–junction usage genome–wide. From 31 866 junctions tested (corresponding to 6579 genes), 2363 junctions in 1320 genes were differentially used in mature mRNA upon WSTF loss (Fig. 5C and Supplementary Table S4). Differential–junction–usage (DJU) events skew toward increased usage in the knockout. Positional analysis of the increased DJU reveals a 5′–3′ bias. Junctions with increased usage cluster near 5′ regions of pre–mRNAs while those with decreased usage concentrate toward 3′ ends (Fig. 5D). This positional bias implies altered RNA polymerase II processivity or disrupted co–transcriptional recruitment of splicing factors in the absence of WSTF, consistent with a chromatin–mediated mechanism for splicing regulation.

These data show that WSTF deficiency reshapes the transcriptome at the isoform and splicing levels in addition to changing gene–level expression. Isoform switching, increased intron retention, altered NMD potential, and positional shifts in splice–junction usage provide mechanistic links between the chromatin state collapse we observed and downstream defects in RNA maturation and stability.

### TCF7L2 exemplifies how WSTF–dependent chromatin changes translate into altered transcriptional outcomes

**Figure 6.**
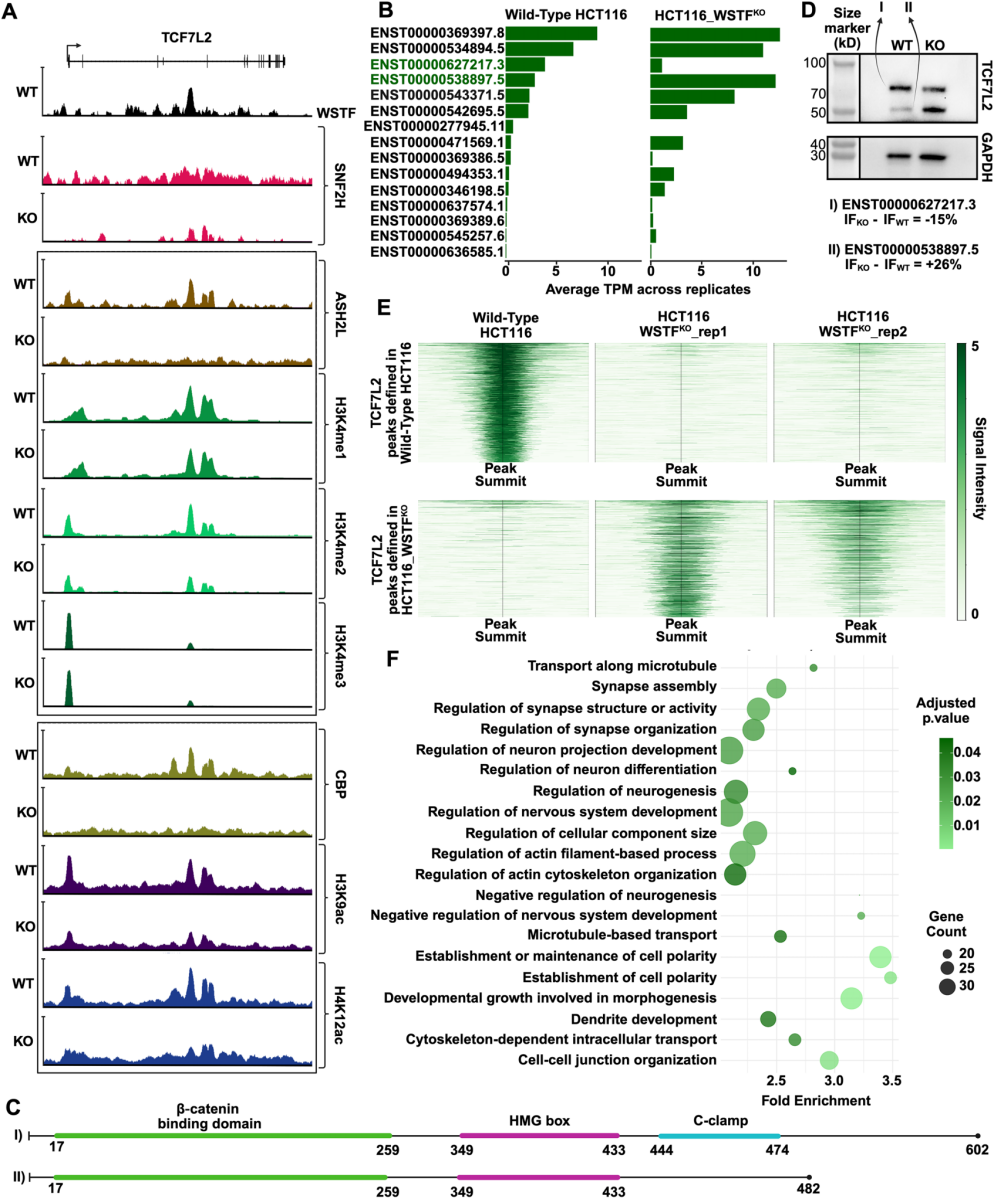
Chromatin-dependent regulation of TCF7L2 splicing and its consequences for Wnt target gene engagement. (**A**) Chromatin landscape of the TCF7L2 locus in HCT116 cells shown as genome browser tracks. Tracks displayed (WT and WSTF^KO^ paired): WSTF, SNF2H, ASH2L, H3K4me1, H3K4me2, H3K4me3, CBP, H3K9ac, and H4K12ac. WSTF and its remodeling partner SNF2H (SMARCA5, the catalytic subunit of the WICH complex) are enriched across the TCF7L2 gene body in WT cells. This enrichment co-occurs with ASH2L and CBP, which are associated with H3K4 methylation and histone acetylation, collectively defining a multi-mark active chromatin state spanning the gene body that is disrupted upon WSTF loss. (**B**) RNA-level TCF7L2 isoform quantification. Bar plot showing mean Salmon TPM averaged across biological replicates for all expressed TCF7L2 transcripts in WT and WSTF^KO^ cells. The shorter TCF7L2 transcript (ENST00000538897.5) increase in abundance in WSTF^KO^ cells. (**C**) Schematic of the two predominant TCF7L2 protein isoforms. Isoform I (ENST00000627217.3) contains the full domain complement: β-catenin-binding domain, HMG box DNA-binding domain, and C-clamp domain (residues 444–474). Isoform II (ENST00000538897.5) lacks the C-clamp domain. IsoformSwitchAnalyzeR analysis identifies a statistically significant isoform fraction (IF) shift: isoform I decreases by 15% (ΔIF = −15%) and isoform II increases by 26% (ΔIF = +26%), confirming at the RNA level the protein-level isoform shift observed in panel (D). (**D**) Western blot of whole-cell lysates from WT and WSTF^KO^ HCT116 cells probed for TCF7L2 and GAPDH (loading control). Two TCF7L2 bands corresponding to the longer (isoform I, ∼75 kD) and shorter (isoform II, ∼55 kD) protein isoforms are visible. The shorter isoform II band is more prominent in WSTF^KO^ cells, corroborating the RNA-level isoform fraction shift. GAPDH confirms equal loading across lanes. (**E**) TCF7L2 chromatin occupancy in WT and WSTF^KO^ cells determined by CUT&RUN. The antibody recognizes an epitope on CTNNB1-binding domain which is shared by both isoforms and therefore reports on the combined TCF7L2 pool. MACS3 peak calling algorithm was applied with significance threshold of *q*-value < 0.01, similar to the rest of the peak calling instances in the current study. Heatmaps show signal intensity at TCF7L2 peaks defined in WT cells (top heatmaps) and at all peaks detected in both WSTF^KO^ biological replicates (bottom heatmaps). TCF7L2 signal is substantially reduced at WT-defined peak sites in both KO replicates, while a distinct set of peaks present in WSTF^KO^ cells is absent from WT. This redistribution of chromatin occupancy is consistent with the predicted change in DNA-binding target selectivity arising from loss of the C-clamp domain in the predominant KO isoform. (**F**) Gene ontology enrichment analysis of TCF7L2 genomic targets defined in wild-type cells.

TCF7L2 encodes a principal TCF/LEF family transcription factor that recruits nuclear β–catenin to Wnt response elements (WREs) and thereby controls canonical Wnt target activation [16]. Because our genome–wide data placed WSTF on active gene bodies and showed coordinated loss of ASH2L/CBP and active histone marks in WSTF^KO^ cells, we asked whether chromatin rewiring at the TCF7L2 locus could directly perturb transcript structure and downstream Wnt signaling. A prominent WSTF binding site lies within the gene body and expectedly colocalizes with ASH2L and CBP in wild–type chromatin. WSTF occupancy at TCF7L2 includes both promoter and gene-body peaks. In WSTF^KO^ chromatin, ASH2L and CBP occupancy at this locus is dramatically reduced and is accompanied by selective depletion of H3K4me2 and H3/H4 acetylation (Fig. 6A). These chromatin changes provide a mechanistic basis for altered co–transcriptional processing at this gene.

TCF7L2 has multiple mRNA isoforms that are expressed in different levels in HCT116 cells and show significantly altered pattern in WSTF^KO^ cells (Fig. 6B). Alternative splicing of TCF7L2 pre-mRNA culminates with protein isoforms showing differential promoter-binding and gene activation potential for β–catenin engagement. Of these isoforms, the canonical one has β–catenin-binding domain, HMG-box, and C-clamp domain (Fig. 6C). Consistent with altered chromatin and predicted effects on mRNA maturation, we observed a shift from the canonical isoform in wild–type cells toward an alternative shorter isoform in WSTF^KO^ cells. The shorter isoform lacks the C–Clamp domain; an auxiliary sequence-selective bipartite DNA binding motif, which contains four highly conserved cysteines along with two clusters of basic amino acids and mediates recognition of 5′-RCCG-3′ motif adjacent to WREs accessing a subset of Wnt targets important in cell proliferation. A subset of Wnt target genes, especially those with weak WREs, have been shown to be selectively regulated by the c-terminal “E-tail” of TCF7 (harboring the C-clamp) and loss of regulation of these genes is suggested to have Wnt-regulated cell phenotypes. β-Catenin capacity to regulate growth in colon cancer cells via modulating the LEF1 promoter is destroyed with mutating the C-clamp of TCF7 [17].

We confirmed that both alternative TCF7L2 transcripts are translated into proteins of the predicted sizes (Fig. 6D). Functionally, isoform shifts in WSTF^KO^ cells mean that the enlarged nuclear pool of stabilized β–catenin encounters a different repertoire of TCF7L2 isoforms with diminished WRE–binding capacity. This altered TCF7L2 population would therefore be expected to reduce or redirect canonical β–catenin genomic target engagement despite elevated nuclear β–catenin levels. Consistent with this prediction, CUT&RUN profiling revealed a marked redistribution of TCF7L2 chromatin occupancy in WSTF–deficient cells (Fig. 6E). TCF7L2 binding was substantially diminished at WT–defined peaks, many corresponding to canonical Wnt effector sites, while a distinct set of peaks emerged uniquely in the knockout, indicating altered DNA–binding target selectivity, driven by loss of the C–clamp domain in the predominant WSTF^KO^ isoform. Gene ontology analysis of WT–defined TCF7L2 targets demonstrated strong enrichment for neuronal and developmental pathways, and these sites showed sharply reduced occupancy in WSTF^KO^ cells (Fig. 6F). Importantly, the de novo peaks that arise in the knockout lacked enrichment for any coherent biological program, demonstrating that the reconfigured TCF7L2 isoform population does not establish an alternative target network but instead disrupts canonical targeting. These findings provide direct mechanistic evidence that isoform remodeling of TCF7L2 under WSTF deficiency produces a redirection, and overall weakening, of β–catenin genomic engagement, explaining how elevated nuclear β–catenin can coexist with a non–canonical transcriptional output.

These observations reconcile the apparent paradox that WSTF loss produces biochemical hyperactivation of Wnt signaling, seen as increased nuclear β–catenin, alongside a non–canonical transcriptional response. WSTF deficiency therefore causes a dual collapse of Wnt signaling: upstream effector hyperactivation coupled with altered downstream transcriptional targeting through isoform remodeling of TCF7L2. This scenario provides a clear example of how WSTF loss–induced locus–specific chromatin changes reprogram regulatory networks and reshape pathway outputs.

### Reduced WSTF dosage produces splicing misregulation in WS patient-derived cells

To assess whether the splicing misregulation we characterize in HCT116-WSTF^KO^ cells reflects a more general consequence of WSTF dosage reduction, we examined differential isoform usage in GM14297 cells derived from a WS patient carrying a hemizygous 7q11.23 deletion (Supplementary Fig. S3A). Although the transcriptional program of this B-lymphoblastoid line differs fundamentally from HCT116 cells, significant changes in isoform fraction were detected: 411 transcripts from 247 genes show significant ΔIF (10%, FDR ≤ 0.05) in the proband relative to both parents (Supplementary Fig. S3B). The affected transcripts are enriched for heterochromatin organization and chromatin regulatory gene ontology terms, mirroring the GO signature of differential junction usage in HCT116-WSTF^KO^ cells. Importantly, the specific isoform switches detected in WS patient cells differ from those in HCT116-WSTF^KO^ cells, consistent with the fundamentally different transcriptional programs and splicing factor repertoires of these two cell types. What is shared is the class of molecular defect: aberrant co-transcriptional splicing affecting chromatin regulatory genes is a conserved consequence of WSTF dosage reduction, whether through complete knockout or hemizygous deletion as occurs in WS.

## Discussion

Our study positions WSTF as a central organizer of an active chromatin signature. Our findings reveal how loss of WSTF leads to misregulation of the chromatin landscape, transcriptional processing, nuclear topology, and signaling network from their canonical regulatory programs.

### WSTF is a protean chromatin factor with a broad regulatory reach

WSTF functions beyond a structural subunit of the WICH complex. WSTF integrates ATP–dependent remodeling with recruitment of histone–modifying activities and co–transcriptional RNA processing. This integration explains why loss of WSTF produces coordinated, not isolated, defects including simultaneous hypoacetylation, selective loss of H3K4 methylation, inappropriate Polycomb engagement, and downstream splicing perturbations. This local scale misregulation is associated with higher order changes. Changes in Lamin–B association indicate that WSTF loss alters higher–order nuclear organization, not just local chromatin marks.

The pervasive isoform switching, intron retention, and altered NMD potential that we observed implicate WSTF-mediated chromatin state as a determinant of transcript architecture. Rather than being a downstream consequence, mis-splicing emerges as a mechanistic mediator that amplifies epigenomic instability. This perspective reframes splicing defects from an epiphenomenon to a central node in Williams Syndrome pathology: altered chromatin leads to altered co–transcriptional processing, resulting in altered isoforms of chromatin factors, which propagates further into chromatin destabilization. Interventions that restore local H3K4 methylation or histone acetylation, or that modulate splicing factor recruitment, therefore have the potential to interrupt this escalatory pathogenic feedback loop.

### Decoupling of Wnt biochemical activation and transcriptional output

Developmentally regulated splicing of TCF7L2 can alter the transcriptional output of the Wnt pathway [18]. TCF7L2 isoforms are major determinants of tissue specific transcriptional output of canonical Wnt signaling. The combination of elevated nuclear β–catenin and altered TCF7L2 isoform repertoire demonstrates that biochemical activation of a pathway does not guarantee canonical transcriptional responses. Chromatin–dependent remodeling of transcription factor isoforms can redirect or blunt pathway outputs even when upstream effectors are abundant. This uncoupling provides a plausible mechanistic link to WS phenotypes and to contexts such as colorectal cancer where β–catenin accumulates in the nucleus. Cells may display biochemical hallmarks of pathway activation while failing to execute the expected gene program. Therapeutic strategies that focus solely on reducing β–catenin levels may therefore be insufficient; restoring correct transcription factor composition or chromatin context could be equally important.

Supporting this idea, the redistribution of TCF7L2 chromatin occupancy in WSTF–deficient cells provides critical insight into how WSTF maintains the fidelity of Wnt/β–catenin signaling. Although WSTF loss produces a markedly enlarged nuclear pool of stabilized β–catenin, isoform remodeling of TCF7L2 disrupts its ability to engage canonical genomic targets and diminishes occupancy at neuronal and developmental regulatory sites. At the same time, the emergence of KO–specific TCF7L2 peaks that lack enrichment for any coherent biological program indicates that the altered isoform repertoire does not establish a compensatory transcriptional network, but instead erodes the specificity of β–catenin–dependent gene regulation. This disconnect between effector abundance and transcriptional output highlights a dual failure of the Wnt pathway under WSTF deficiency: biochemical hyperactivation coupled with loss of appropriate genomic targeting. These results position TCF7L2 isoform remodeling as a central conduit through which WSTF–dependent chromatin architecture shapes downstream signaling outcomes and, when perturbed, contributes to the regulatory miswiring characteristic of Williams syndrome. These TCF7L2–dependent defects indicate that WSTF loss disrupts Wnt signaling downstream of β–catenin, a mechanism that complements receptor–level dysregulation caused by FZD9 haploinsufficiency in Williams syndrome.

### WSTF and FZD9 converge on Wnt/β-catenin signaling in Williams syndrome

Our findings identify a mechanistic link between WSTF loss and Wnt/β-catenin hyperactivation through two routes: constitutive nuclear accumulation of active β-catenin, and isoform switching of TCF7L2 toward a shorter form lacking the C-clamp domain, which alters genomic target engagement. An important context for these observations is that FZD9, which encodes Frizzled-9, a Wnt co-receptor, is also deleted in the WS microdeletion interval. Prior work has demonstrated that FZD9 loss contributes independently to Wnt pathway dysregulation in WS [19–21]. This creates a convergent mechanism: FZD9 haploinsufficiency alters receptor-level pathway activation, while WSTF loss disrupts the chromatin and isoform landscape of the key transcriptional effector TCF7L2 and drives constitutive β-catenin nuclear accumulation independent of exogenous ligand. Co-deletion of both genes in WS patients therefore likely produces compounded Wnt pathway dysregulation through complementary mechanisms, which may amplify the cardiovascular and neurodevelopmental phenotypes characteristic of the syndrome. Testing whether FZD9 and WSTF loss act additively or synergistically on Wnt target gene programs in WS-relevant cell types represents an important direction for future work.

The present study uses homozygous WSTF knockout cells to maximize the chromatin and transcriptome signal for characterizing WSTF-dependent regulatory relationships. Heterozygous HCT116-WSTF⁺/⁻ cells, which better model the single-allele loss that occurs in WS patients, were generated as intermediates during the CRISPR editing strategy and are available for analysis. Comparing homozygous KO and heterozygous phenotypes will be important for determining which effects are dosage-sensitive versus requiring complete WSTF loss, and for calibrating the relevance of our findings to WS haploinsufficiency. Similarly, characterizing WSTF chromatin occupancy patterns in WS-derived cells and in disease-relevant lineages such as neural crest and cardiac progenitors will be essential for determining which WSTF-dependent regulatory relationships identified here are conserved in the tissues most affected in WS.

### Therapeutic opportunities

The multilayered defects we describe point to several experimentally tractable interventions for Williams Syndrome and other diseases including locus–specific restoration of H3K4me2/acetylation, forced expression of canonical transcription factor isoforms (e.g. TCF7L2), or targeted modulation of splicing factors. Testing these interventions will distinguish which defects are primary and which are secondary, suggesting which downstream vulnerabilities are most amenable to rescue. Our reliance on HCT116 cells provides experimental tractability but limits tissue specificity. The chromatin and splicing landscapes of neural crest cells or cardiomyocytes may modulate the magnitude or identity of WSTF–dependent effects. Extending analyses to primary or induced neural crest and cardiac lineages, and applying orthogonal approaches (proteomics of promoter complexes, single–molecule Pol II kinetics, and locus–specific epigenetic editing), will be essential to validate generality, establish causality, and prioritize therapeutic targets. From a translational perspective, targeting splicing modulators or chromatin “writers” may offer routes to mitigate developmental or oncogenic consequences of WSTF deficiency.

## Conclusion

WSTF loss exposes a foundational principle of genome regulation: active chromatin signatures are multiplexed platforms that coordinate remodeling, histone modification, and RNA processing to preserve transcript integrity and faithful signaling outputs. Disruption of a single organizing factor can therefore cascade into diverse molecular and cellular pathologies. Recognizing and targeting the nodes that propagate these cascades, including chromatin writers, splicing regulators, and transcription factor isoform balance, offers a focused strategy to correct the downstream consequences of chromatin factor dysfunction in developmental disorders and cancer.

## Supplementary Material

gkag602_Supplemental_Files

## Data Availability

All sequencing data used in the current study for chromatin profiling and transcriptome analysis have been deposited to the NCBI's Gene Expression Omnibus (GEO) database under accession numbers GSE314624 and GSE314625.
