# Supplementary material for: WSTF deficiency reprograms regulatory networks by linking locus–specific chromatin remodeling to altered isoform expression and misdirected signaling
Source: Nucleic Acids Res. 2026 Jun 8;54(11):gkag602. doi: 10.1093/nar/gkag602 (PMC13244151; doi:10.1093/nar/gkag602)
Supplement: gkag602_Supplemental_Files [file gkag602_supplemental_files.zip › Supplementary Movie 1.docx]

**Supplementary Movie 1.** Three-dimensional reconstruction of a single HCT116‑WSTF-KO nucleus imaged at 100× magnification. The movie comprises 50 z‑stacks spanning approximately 10 µm and shows a merged view of DAPI (blue) and FITC (β‑catenin, green) channels. A single intranuclear β‑catenin aggregation focus is visible, occupying nuclear space without apparent association with DNA.
