# Supplementary material for: WSTF deficiency reprograms regulatory networks by linking locus–specific chromatin remodeling to altered isoform expression and misdirected signaling
Source: Nucleic Acids Res. 2026 Jun 8;54(11):gkag602. doi: 10.1093/nar/gkag602 (PMC13244151; doi:10.1093/nar/gkag602)
Supplement: gkag602_Supplemental_Files [file gkag602_supplemental_files.zip › Revised_Supplementary_Material_editable.doc]

**Supplementary Table 1. List antibodies used in the current study.**

**Supplementary Table 2. List of differentially expressed genes in HCT116-WSTF^KO^.** The list is the result of differential gene expression analysis obtained from DESeq2 based analysis or RNAseq data.

**Supplementary Table 3. List of differentially expressed isoforms in HCT116-WSTF^KO^.**

**Supplementary Table 4. List of splice junctions with differential usage in HCT116-WSTF^KO^.**

**Supplementary Table 5. Splice-site–anchored ASH2L and CBP signal at donor and acceptor sites, stratified by exon position. (A)** ASH2L and CBP CUT&RUN normalized signal was mapped around splice sites in a strand-aware, splice-site–anchored coordinate system (±200 bp around the splice coordinate; 10-bp bins). Donor sites correspond to the 5′ splice site (exon end) and acceptor sites correspond to the 3′ splice site (exon start). Exon-position strata were defined as donor-first (end of first exons), donor-internal (end of internal exons), acceptor-internal (start of internal exons), and acceptor-last (start of last exons). Local boundary signal (peak_local) was quantified as the mean signal within ±20 bp of the splice coordinate, computed as the average of exon-side and intron-side edge signals: peak_local = (exon_edge + intron_edge)/2. Exon-side and intron-side edge signals were computed from the two 10-bp bins immediately adjacent to the splice coordinate. Sites were restricted to those with nonzero local signal in WT or KO (peak_local_WT > 0 or peak_local_KO > 0) to avoid domination by all-zero loci. **(B)** Edge asymmetry was quantified as edge_delta = exon_edge − intron_edge; WT→KO changes were assessed with paired Wilcoxon signed-rank tests across matched sites within each stratum. “Donor>Acceptor p (WT/KO)” reports the one-sided Wilcoxon rank-sum test (alternative = donor > acceptor) comparing donor vs acceptor peak_local distributions within genotype.

**Supplementary Table 6. Exon-position contrasts disentangling apparent junction enrichment from exon-end contributions.** Using the same splice-site–anchored matrices and filtering as in Tables 6a and 6b (±200 bp window; 10-bp bins; retaining sites with peak_local > 0 in WT or KO), three targeted comparisons were performed to directly address the possibility that “junction peaks” could reflect preferential enrichment at 3′ exon ends or be dominated by gene-end exons. “Median A” and “Median B” are the medians of peak_local in the two groups being compared; “Median (A minus B)” is their difference. For donor.internal > acceptor.internal, the Wilcoxon test used a one-sided alternative (donor > acceptor) to explicitly test for a donor-side bias on internal exons. The donor.first vs donor.internal and acceptor.last vs acceptor.internal comparisons used two-sided Wilcoxon tests to quantify 5′-end (first exon) and 3′-end (last exon) contributions, respectively, within each genotype.

**Supplementary Figure 1. CRISPR/Cas9‑mediated complete deletion of WSTF locus. (A)** Immunofluorescence of interphase wild‑type HCT116 and HCT116‑WSTF knockout (HCT116‑WSTF KO) cells stained with anti‑WSTF, and anti‑tubulin primary antibodies followed by fluorescently labeled secondary antibodies. Nuclei are counterstained with DAPI. **(B)** Schematic showing annealing locations of guide RNAs (gRNAs) and PCR primers used to delete the genomic interval and to screen edited clones. The gRNA5‑1/gRNA3‑1 pair was used to delete the first allele and screened with the F1/R1 primer set; the gRNA5‑2/gRNA3‑2 pair was used to delete the second allele and screened with the F2/R2 primer set. **(C)** Screening PCR on genomic DNA from wild‑type and edited HCT116 genomes. No PCR product is obtained from wild‑type DNA because the distance between primers is ~80 kb (beyond standard PCR capacity); following successful deletion, primer annealing sites are brought into close proximity and a diagnostic PCR product is produced. **(D)** Western blot of biological replicates from wild‑type HCT116 and HCT116‑WSTF KO cells demonstrate complete loss of WSTF protein in the edited lines.

**Supplementary Figure 2. Nuclear β-catenin quantification and distribution analysis.** Illustration of the cell segmentation and signal quantification approach used for Fig. 2C–D. For each cell, three measurements were collected from the non-phospho-Ser45 β-catenin immunofluorescence channel under identical imaging conditions: (i) local background (sampled in an extracellular region adjacent to the cell), (ii) whole-cell integrated density (bounded by the cell perimeter defined by β-catenin staining), and (iii) nuclear integrated density (bounded by the DAPI mask). Background-corrected cytoplasmic signal was derived as (whole-cell integrated density) − (nuclear integrated density), and the nuclear-to-cytoplasmic (N/C) ratio was computed per cell. This approach isolates the nuclear enrichment of active β-catenin independently of variation in total cellular protein levels. Representative segmentation outlines are shown for a WT and a WSTF^KO^ cell.

**Supplementary Figure 3. Differential isoform expression detected in WS patient. (A)** Schematic of the Williams syndrome critical region on chromosome 7 that is deleted in affected individuals along with the pedigree of the Williams syndrome family analyzed in this study. **(B)** Differential isoform expression in the B‑cell transcriptome of the Williams syndrome proband (GM14297). Physiologically relevant genes, including chromatin remodelers and B‑cell–specific genes, are highlighted.
